# Clinical, Radiological, and Functional Evaluations of the Anterior-to-Psoas Lumbar Interbody Fusion Approach With Posterior Decompression and Osteotomy for Treating Patients With Adult Spinal Deformity: A Retrospective Study

**DOI:** 10.7759/cureus.77138

**Published:** 2025-01-08

**Authors:** Joshua P Herzog, Amy Rosenthal, Dakshith Ragupathi, Erin L Brown, Brandon S Bucklen

**Affiliations:** 1 Spine Surgery, OrthoVirginia, Richmond, USA; 2 Clinical Affairs, Globus Medical Inc., Audubon, USA; 3 Clinical Operations, Globus Medical Inc., Audubon, USA; 4 Research, Globus Medical Inc., Audubon, USA

**Keywords:** adult spinal deformity, anterior-to-psoas approach, expandable interbody spacers, lumbar interbody fusion, minimally invasive surgery

## Abstract

Objective: Degenerative adult spinal deformity (ASD) is a prevalent disease in the elderly population. Treating it typically requires an extensive surgical intervention. This study aimed to assess the use of expandable spacers for multi-level anterior-to-psoas lumbar interbody fusion (ATP-LIF) along with posterior direct decompression and osteotomy to treat patients with degenerative ASD.

Method: This was a single-center retrospective study of ASD patients (anyone undergoing fusion procedure for four or more spinal levels) undergoing two-stage surgery with expandable interbody spacers with a minimum follow-up period of around 12 months between November 2019 and June 2021. A total of 20 patients were enrolled in this study (15 patients = four-level fusion, five patients = five-level fusion). Exclusion criteria included <18 years of age, pregnancy, tumor, and trauma patients. Demographic, surgical, radiographic, complications, and patient-reported outcomes (PROs) were collected.

Results: Of the 20 patients included (mean age = 68.1 ± 9.0 years, mean body mass index = 30.5 ± 7.4 kg/m^2^, 12 males and eight females), surgical data showed a mean total operation time of 315 ± 180.6 mins and mean total blood loss of 638.8 ± 37.6 mL. At 12 months, 18/20 patients returned for follow-up. There was a significant reduction in the mean visual analog scale (VAS) back pain scores (Δ = 2.1, p < 0.05), an increase in the 12-item short-form health survey (SF-12) (Δ = 5.6, p < 0.05), and improvement in pelvic incidence-lumbar lordosis (PI-LL) (Δ = 15.8°, p < 0.05) at the final follow-up, as compared with the preoperative baseline. Moreover, at 12 months follow-up, radiographic data showed 100% fusion rate (18/18), lumbar lordosis (Δ = 13.9°), and disc height improvement (Δ = 4.1 mm), as well as a reduction in the coronal cobb angle (Δ = 6.9°) as compared to the preoperative baseline.

Conclusion: Two-stage ATP-LIF with expandable spacers along with posterior direct decompression and Ponte osteotomy is a viable minimally invasive treatment for patients with ASD. This was evidenced by similar surgical outcomes to pedicle subtraction osteotomy, improvements in PROs, restoration of PI-LL, high fusion rates, and a significant increase in disc height.

## Introduction

A novel iteration of the anterior lumbar interbody fusion (ALIF) approach was first described by Mayer et al. in 1997 [1]. It made use of the extraperitoneal space between the abdominal aorta and psoas muscles such that the anterolateral surface of the intervertebral disc space could be approached with minimal disruption to the patient’s anatomy [2]. This minimal invasive approach was later coined by Silvestre et al. as oblique lumbar interbody fusion (OLIF) in 2012 [3]. Over time, this approach has gained more widespread acceptance as compared to traditional approaches like posterior lumbar interbody fusion (PLIF), lateral lumbar interbody fusion (LLIF), and ALIF. This may be due to OLIF's superior trajectory in terms of avoiding retraction of the thecal sac and nerve roots (in PLIF), the great vessels, and the hypogastric plexus (in ALIF) [4-8]. Moreover, unlike the lateral lumbar interbody fusion (LLIF), it avoids the psoas muscle and the lumbar plexus. The avoidance of critical nerves, blood vessels, and soft tissues with the OLIF approach has been theorized to result in lower (or similar) rates of approach-related complications, as compared to traditional methods [9-12]. For these reasons, the use of OLIF (also referred to as anterior to psoas lumbar interbody fusion (ATP-LIF) in this study) is being adopted for treating adult spinal deformity (ASD) patients in a minimally invasive manner [13-17].

Historically, ASD cases were performed entirely through a posterior approach. It involved performing a very disruptive posterior subtraction osteotomy (PSO) for sagittal alignment correction, which has been found to be associated with a high complication rate and blood loss. Recently, ATP-LIF has been adopted for the minimally invasive treatment of ASD patients [13-17]. This modified approach provides clear access for anterior longitudinal ligament (ALL) resection, allowing for greater lumbar lordosis correction. Moreover, it provides a minimally disruptive corridor for discectomy and interbody placement as compared to the traditional methods for lumbar interbody fusion, thus avoiding complications such as dural tears and nerve root injuries [18]. The use of expandable spacers while performing ATP-LIF allows for easy lateral spacer placement (due to low starting height during insertion) in the disc space, thereby enabling a smooth experience while positioning the spacer within the disc space. Expandable spacers have also been shown to better preserve the end-plates than static cages due to less impaction needed during insertion into the disc space, thus yielding higher fusion rates [19-21]. The objective of this study was to investigate the clinical use of expandable spacers for ATP-LIF in conjunction with a Ponte osteotomy to treat patients with ASD.

## Materials and methods

Study design

This was a retrospective study of patients undergoing a two-stage correction of ASD using expandable interbody spacers through the anterior-to-psoas approach along with Ponte osteotomy and supplemental posterior fixation between November 2019 and June 2021. The criteria for being considered an ASD was any patient who underwent ATP-LIF for four or more levels. A total of 20 nonconsecutive patients met the inclusion and exclusion criteria (details mentioned in the next couple of sentences) and were included for analysis in this study. A board-certified orthopedic spine surgeon operated on all patients at a single surgery center (Johnston-Willis Hospital, Richmond, VA). The study included any >18-year-old patient who underwent ATP-LIF using RISE®-L expandable spacers (Globus Medical, Audubon, PA) as part of an anterior-column realignment in a two-stage spinal deformity correction to treat lumbosacral degenerative ASD. The exclusion criteria for the study were any patient who met either one or more of the following at the time of the surgery: (1) pregnant, (2) any tumor, (3) trauma, and (4) any prior fusion surgery at the treatment levels. Twenty patients met the inclusion criteria, and none were excluded from the study. A global IRB approval from the Chippenham and Johnston-Willis Hospital Institutional Review Board (CJW IRB) was obtained before conducting the retrospective chart review (approval no. 2022-004-CJWIRB). The minimum follow-up period for this study was 12 months.

Outcomes measured

This study involved retrospective collection of surgical data (total skin-to-skin operation time for each stage in minutes, estimated blood loss in mL and intraoperative complications (IOC)), postoperative data (length of stay, postoperative complication rate, and revision surgery rate), radiographic data (pelvic incidence, pelvic tilt, sacral slope, lumbar lordosis, coronal Cobb angle, fusion rate, disc and neuroforamen heights), and PROs (VAS (visual analog scale) pain score, ODI (Oswestry disability index), and SF-12 (12-item short form survey)).

Data collection

**Figure 1:**
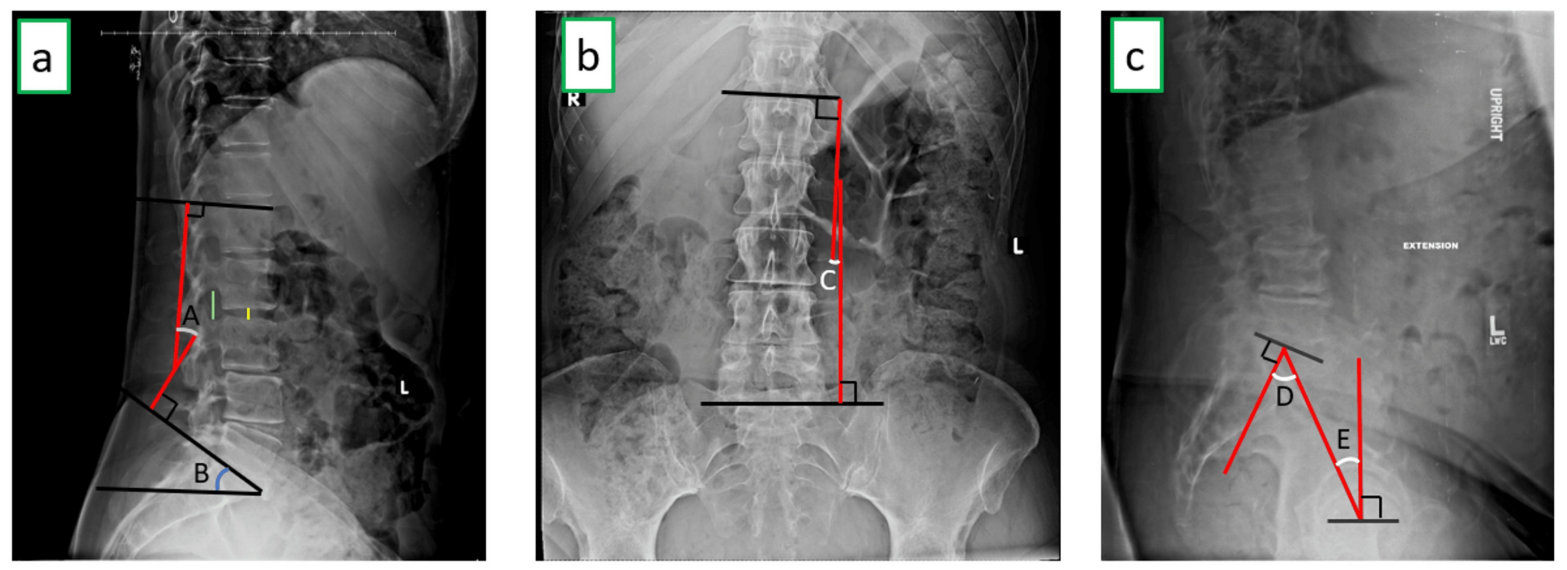
Illustration of the X-ray measurements a (left) shows a lateral lumbar X-ray measuring lumbar lordosis - LL (A), sacral slope – SS (B), disc height – DH (yellow line) and neuroforamen height - NFH (green line). b (middle) shows an AP image of the lumbar spine used to measure the coronal Cobb angle – CC (C). c (right) shows lateral X-ray used to measure pelvic incidence - PI (D) and pelvic tilt - PT (E).

All data collection was done only after getting a global IRB approval that exempted informed consent due to the nature of this study being a retrospective chart review. In addition, all protected health information (PHI) was removed and only deidentified data were collected in this study. Surgical and postoperative data were collected through surgical and postoperative visit notes, and the minimum follow-up period was 12 months. Radiographic data were derived from AP, lateral, and flexion-extension lumbar X-rays. They were taken at the following time points: preoperative, six months, and 12 months. Figure 1a-1c shows details on radiographic measurements. Fusion was defined as a radiograph (AP and lateral X-rays) demonstrating evidence of bridging bone and absence of radiolucency around the spacer and <3-degree movement on flexion-extension X-ray [22]. The radiographic measurements were done by a single surgeon.

Statistical analysis

All surgical and postoperative data are displayed in tabular format and include descriptive statistics such as count, percentage, arithmetic mean, and sample standard deviation. They were performed using Excel® 2019 (Microsoft, Redmond, WA). All radiographic and PRO data were compared between preoperative and postoperative time points with the Wilcoxon signed-rank test using IBM SPSS Statistics for Windows, version 20.0 (released 2011, IBM Corp., Armonk, NY). Based on a literature search, minimal clinically important difference (MCID) was defined as changes in VAS pain score of two points and ODI of 10 points [23].

Surgical technique

The surgery was performed in two stages (Day 1 = Stage 1 and Day 2 = Stage 2). Stage 1 included the placement of the spacers and indirect decompression, whereas the next day was Stage 2, which included the placement of posterior fixation instruments, Ponte osteotomy, and direct decompression. There were no recorded complications between stages. For Stage 1, the patient was placed in a lateral position, and intraoperative fluoroscopy was used for stereotactic guidance. Based on fluoroscopic images, projected disc spaces are marked on the patient’s skin. Then, a McBurney incision (2-3 inches) is made such that it aims toward the anterior margin of the target disc space. Blunt finger dissection was performed to get past the abdominal wall muscles, till the transversalis fascia is seen and has entered the peritoneal space. The peritoneal cavity and vascular structures are retracted anteriorly, whereas the psoas muscle and retroperitoneum is retracted posteriorly, such that the disc space can be clearly seen for discectomy. Pituitary and Kerrison rongeurs are used to remove and prepare the end plates. Partial anterior longitudinal ligament resection (PALLR) was performed as mentioned in Jeon et al. [13]. A RISE®-L expandable spacer (Globus Medical Inc, Audubon, Pa) packed with autograft (harvest from the iliac crest) was positioned laterally in the disc space and expanded till the desired disc height was achieved. The retractors were removed, and the abdominal structures and psoas returned back to their normal position.

Stage 2 involved an open surgery approach. The patient was placed in the prone position and a midline incision was made on the spinous process of the affected vertebrae. The soft tissue was dissected and a retractor was used for clear visualization of the spine. Direct decompression at the affected levels was performed with laminectomies, and removal of bony spurs, hypertrophic facets, and ligamentum flavum using a high-speed burr, pituitary, or Kerrison rongeur. Ponte osteotomies were also performed to provide adequate lordosis correction. Pedicle screws were inserted using an open freehand technique and a rod was placed (CREO® Threaded Stabilization System, Globus Medical) over to provide posterior fixation. Retractors were removed, and the incision was closed with suture and dressing.

## Results

Out of 20 patients included in this study, 18 patients returned for the 12-month follow-up visit (mean follow-up = 12.1 months, ranging from 10 to 14 months) and two were lost to follow-up (2/20 = 10% loss). The number of operated levels was 4 (n = 15, 75%) and 5 (n = 5, 25%). Surgical data showed a mean Stage 1 skin-to-skin operation time (OT) of 134.9 ± 28.2 minutes and a mean Stage 2 skin-to-skin operation time (OT) of 180.1 ± 47 minutes, and the mean combined skin-to-skin total operation time (TOT) was 315 ± 180.6 minutes. The mean estimated blood loss (EBL) for Stage 1 was 111.3 ± 94.1 mL and Stage 2 was 527.5 ± 267.3 mL, with the total mean estimated blood loss (TEBL) of 638.8 ± 37.6 mL. In addition, this cohort showed 5% (n = 1) intraoperative complications. The average length of stay in hospital was 5.4 ± 3.5 days. See Table 1 for a consolidated view of the demographics and surgical data.

**Table 1:** Patient demographics and surgical data

Characteristic	Descriptive statistics (mean ± Std)
Total patients = 20	
Age at surgery (years)	68.1 ± 9.0 years
Male sex, n (%)	12 (60.0 %)
BMI at surgery (kg/m^2^)	30.5 ± 7.4 kg/m^2^
Smoking status, n (%)	
Current	3 (15.0 %)
Electronic	1 (5.0 %)
Past	8 (40.0 %)
Never	8 (40.0 %)
Comorbidities, n (%) (non-mutually exclusive)	
Osteoporosis	2 (10.0 %)
Diabetes	4 (20.0 %)
Cardiac problems	19 (95.0 %)
Pulmonary	9 (45.0 %)
Endocrine	5 (25.0 %)
Neurological	3 (15.0 %)
Gastrointestinal	10 (50.0 %)
Genitourinary	7 (35.0 %)
Cancer or immune disorder	8 (40.0 %)
Psychological	7 (35.0 %)
ASA Scores, n (%)	
2 – Mild systemic disease	4 (20.0 %)
3 – Severe systemic disease	16 (80.0 %)
# of operated levels of surgery	
4	15 (75 %)
5	5 (25 %)
Total estimated blood loss (mL), mean	638.8 ± 180.6 mL
Stage 1	111.3 ± 94.1 mL
Stage 2	527.5 ± 267.3 mL
Skin-to-skin total operation time (mins), mean (SD)	315 ± 37.6 mins
Stage 1	134.9 ± 28.2 mins
Stage 2	180.1 ± 47 mins
Intraoperative complications, n (%)	1 (5 %)
Length of stay in hospital (days): mean ± std {Range}	5.4 ± 3.5 {2-14} days
Rehabilitation nights (days): mean ± SD {Range}	3.1 ± 6.2 {0-18} days

**Figure 2:**
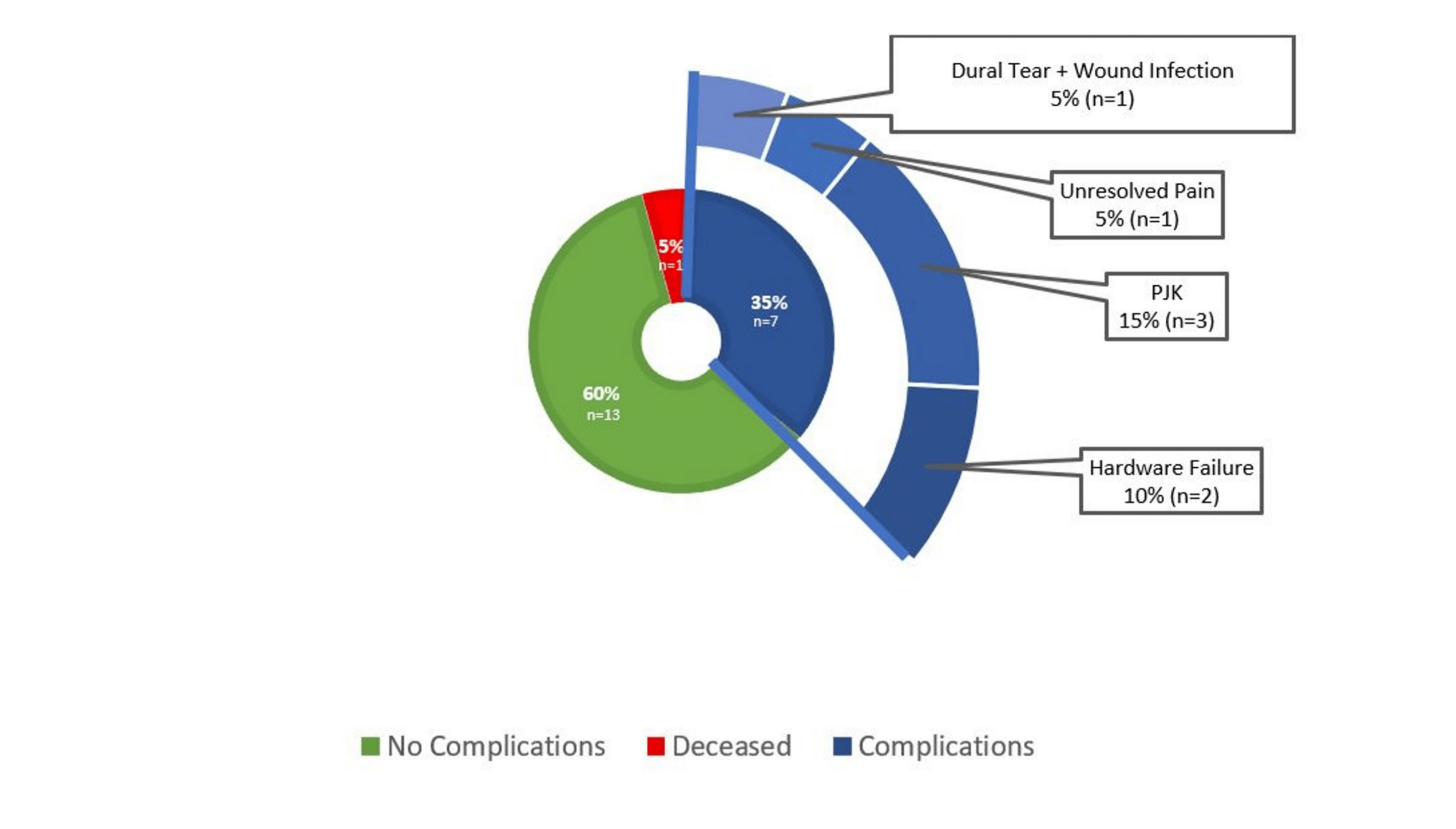
12-month postoperative complication and revision surgery data

Postoperative data showed that 35% (n = 7/20) developed complications and required revision surgery (the mean time for revision surgery was nine months from index surgery). Moreover, one patient died postoperatively (5%, n = 1/20) due to a cardiac-related problem. Reasons for revision surgery were as follows: proximal junction kyphosis (n = 3, 15%), hardware failure (n = 2, 10%), unresolved pain (n = 1, 5%), and wound infection with dural tear (n = 1, 5%). See Figure 2 for a consolidated view of the complication data.

**Figure 3:**
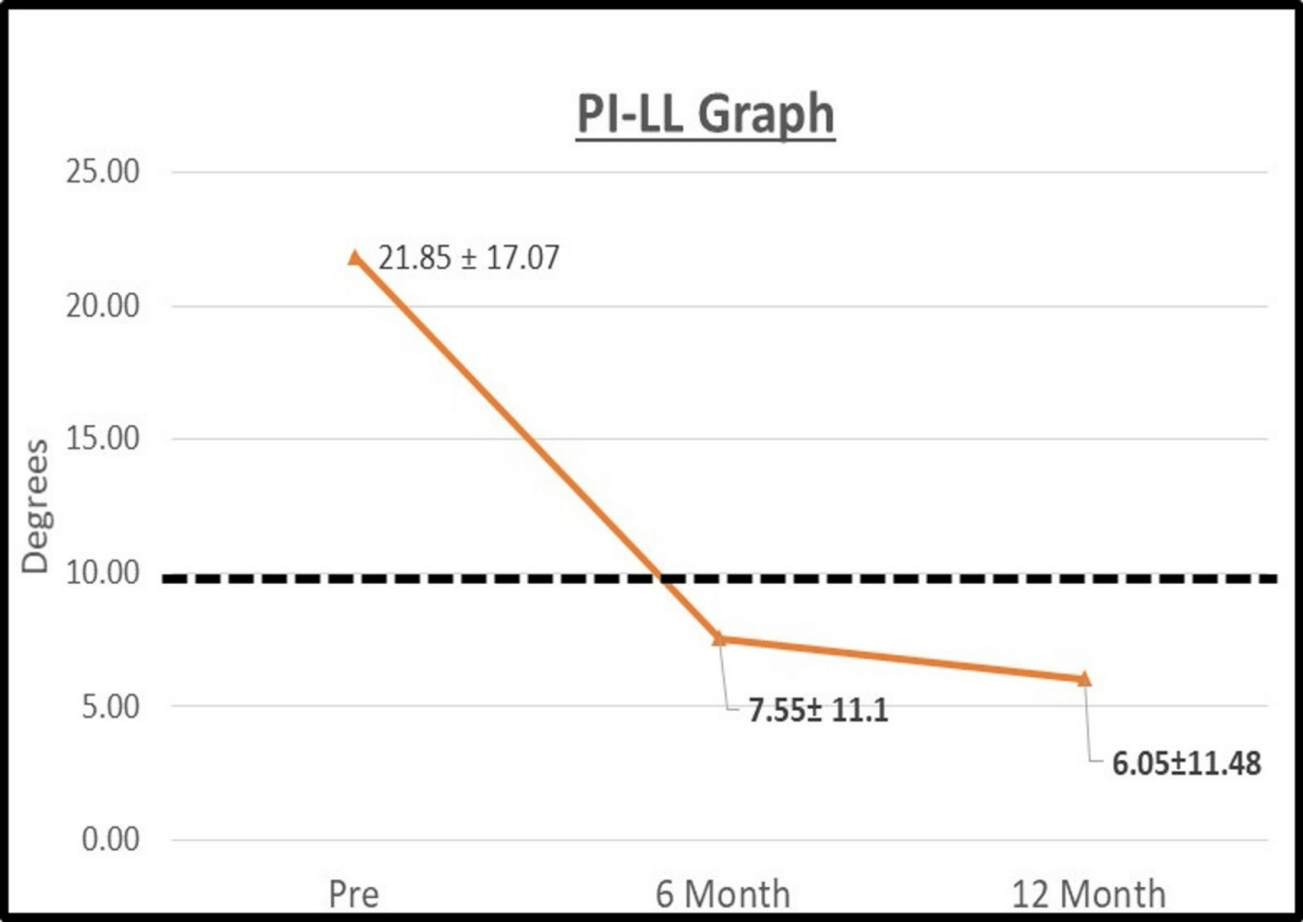
Pelvic incidence-lumbar lordosis (PI-LL) mismatch between the preoperative and postoperative timepoints The figure shows the average and standard deviation of the pelvic incidence-lumbar lordosis (PI-LL) mismatch for each time point. Bold lettering indicates p < 0.05 when compared to preoperative data. The black dashed horizontal line indicating PI-LL mismatch average dropping below 10 degrees, known to be ideal for sagittal alignment correction [24].

**Figure 4:**
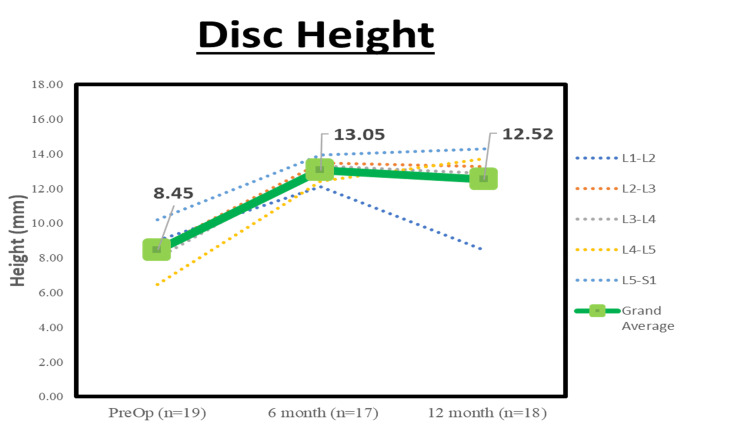
Mean disc height at preoperative and postoperative timepoints Average disc height of per segmental level and total per patient for each time point. There was statistically significant improvement (p < 0.05) between preoperative and all postoperative follow-up visits.

Radiographic parameters are displayed in Table 2. At the 12-month follow-up, there was statistically significant (p < 0.05) improvement in ΔLL of 13.9° and Δ coronal Cobb of 6.9°. The fusion rate was 5.9% at six months and 100% (18/18 patients) at 12 months. There was a statistically significant reduction in PI-LL at both six months postoperative (Δ = 14.3°) and 12 months postoperative (Δ = 15.8°) (see Figure 3). The overall increase in NFH was 1.8 mm between the 12 months postoperative and preoperative (see Table 3 for the segmental changes). In addition, a statistically significant increase in the overall disc height of Δ= 4.6 mm at six months postoperative and Δ= 4.1 mm at 12 months postoperative was seen when compared with preoperative data (see Figure 4).

**Table 2:** Consolidated view of all radiographic measurements * = p < 0.05 between baseline (preoperative) and postoperative visits. PI-LL: pelvic incidence-lumbar lordosis

Measurements	Preoperative (n = 19)	Six months postoperative (n = 17)	12 months postoperative (n = 18)
Pelvic incidence (°)	56.4 ± 13.3	55.2 ± 13.6	55.1 ± 16.0
Pelvic tilt (°)	24.3 ± 11.3	22.1 ± 7.6	20.1 ± 9.9
Sacral slope (°)	31.4 ± 8.7	34.2 ± 9.5	35.3 ± 10.6
Lumbar lordosis (°)	34.5 ± 12.0	46.3 ± 14.0 *	48.4 ± 13.8 *
Coronal Cobb angle (°)	16.3 ± 8.0	11.5 ± 5.2	9.4 ± 3.2 *
PI-LL (°)	21.9 ± 17.1	7.6 ± 11.1 *	6.1 ± 11.5 *
Fusion rate (%)	.8	5.9%	100.0 %

**Table 3:** Descriptive statistics of the neuroforaminal height (mm) * = p < 0.05 between the baseline (preoperative) and postoperative visits

Segmental levels	Preoperative (n = 19)	Six months postoperative (n = 17)	12 months postoperative (n = 18)
L1-L2	20.1 ± 4.3	22.2 ± 3.82	22.4 ± 6.7
L2-L3	18.8 ± 5.6	22.1 ± 4.7	21.6 ± 4.2
L3-L4	18.0 ± 5.7	20.1 ± 5.7	20.2 ± 4.5
L4-L5	17.3 ± 5.9	20.5 ± 6.0	20.8 ± 4.9
L5-S1	19.6 ± 17.8	16.8 ± 4.3	18.1 ± 4.4
Grand average	18.8 ± 5.7	20.3 ± 3.6	20.6 ± 3.2

PROs had a minimum follow-up of 12 months. The cohort showed an average VAS back pain score improvement of 2.1 with statistical significance at both six and >12-month follow-ups. The average ODI improved from 22.6 (preoperative) to 17.3 (six months postoperative) to 18.5 (>12 months postoperative). There were 50% (9/18) of patients who achieved MCID for VAS (i.e., ΔVAS ≥ 2) and 40% (4/10) of patients who achieved MCID for ODI by the 12-month follow-up. The mean SF-12 improved significantly between the preoperative and last follow-up (preoperative = 24.3 vs. six-month postoperative = 26.3 vs. >12-month postoperative = 29.9). See Table 4 for a consolidated view of the PRO results.

**Table 4:** Consolidated view of the patient-reported outcomes (PROs) * = p < 0.05 between the baseline (preoperative) and postoperative visits. VAS = visual analog scale, ODI = Oswestry disability index, SF-12 = 12-item short form health survey

PRO	Preoperative	Six months postoperative	>12 months postoperative
VAS (visual analog scale) for back pain	6.1 ± 2.6 (n = 20)	2.7 ± 2.6 * (n = 17)	4.0 ± 3.5 * (n = 18)
ODI (Oswestry disability index)	22.6 ± 6.0 (n = 17)	17.3 ± 11.8 (n = 10)	18.5 ± 17.1 (n = 10)
SF-12 (12-item short form health survey)	24.3 ± 3.0 (n = 17)	26.3 ± 6.00 (n = 10)	29.9 ± 5.9 * (n = 9)

An example case

A male, 64-year-old patient with a BMI of 37.4 (obese) and multiple comorbidities was diagnosed with degenerative scoliosis and lumbar radiculopathy. He underwent a two-stage procedure as mentioned in this study from L2-S1. The patient experienced no intraoperative or postoperative complications. At the 12-month follow-up, the patient showed a PI-LL of 9 (vs. 24 at preoperative), coronal Cobb angle of 11 (vs. 27 at preoperative), mean neuroforaminal height of 21 mm (vs. 15.2 mm at preoperative), and mean disc height of 19.6 mm (vs. 53.0 mm at preoperative). Moreover, the PROs showed a 12-month VAS back pain score of 1 (vs. 5 at preoperative), ODI of 8 (vs. 25 at preoperative), and SF-12 of 32 (vs. 22 at preoperative). Lastly, the patient achieved MCID for all the PROs.

## Discussion

ATP-LIF is a minimally invasive approach to perform interbody fusion. Expandable spacers are designed to be easily positioned within the disc space and cause minimal disruption to the endplates. The combination of using expandable spacers for ATP-LIF to provide sagittal and coronal correction for ASD patients in a minimally invasive manner has not been extensively studied. Therefore, this study aimed to look at the viability of using expandable spacers for ATP-LIF to restore disc height and coronal alignment, by evaluating the number of sagittal corrections achieved by performing a two-staged procedure that includes a posterior and an anterior-to-psoas approach. 

Reviewing the surgical data, the average TEBL and TOT were 638.8 mL and 315 minutes, respectively. These were in line with existing literature on Ponte osteotomy, which shows blood loss to be within 299.86-1377.72 mL and operation time within 213.4-322.4 minutes [25]. It indicates that this study's two-staged approach yields similar surgical outcomes as when compared to a well-established Ponte osteotomy procedure. Furthermore, this cohort showed that only one patient developed an intraoperative complication, and it was a dural tear, an expected complication for the procedure in this study. The low intraoperative complication rate may be due to the less invasive nature of this surgery, surgeon skills and experience, or just a potential limitation of a single-site study. Some highlightable steps taken to minimize intraoperative complications (like nerve and vascular injuries) were identifying and protecting blood vessels and frequent use of radiographic imaging to better visualize the trajectory. The postoperative complication rate at 12-month follow-up was 35% in the current study. This was above the typical complication rate shown in patients undergoing Ponte osteotomy, which ranged from 2.5% to 32.4% [26]. Half of the revisions in this study were due to proximal junctional kyphosis (42.9%, n = 3/7), which is a common complication in patients who have undergone multi-level fusion surgeries and are unrelated to approach or interbody devices. Other indications for revision surgery were due to wound infection, revision of posterior fixation components, and unsettled pain. In addition, this study cohort presented with multiple pre-existing comorbidities, and this may be the reason for such a high revision rate.

A systematic review and meta-analysis performed by Zhu et al. on OLIF with posterior osteotomy for ASD (a similar surgical technique as this study) showed improvements in coronal Cobb angle ranging from 5.8° to 19° [27], similar to the results from this study with a mean coronal Cobb angle improvement of 6.9°. The placement of the spacer in a lateral position may have helped with improving coronal Cobb. It was easily placed in a lateral position using an oblique approach due to the maneuverability created by the low starting height of the expandable spacer during insertion. This study also showed a statistically significant increase in average global lordosis (ΔLL = 13.9°), which aligns with published literature with an average lordotic change of 13.5° [13]. At the 12-month follow-up, a statistically significant PI-LL mismatch change of 15.8° was seen in this study, and it was slightly higher than the current literature on OLIF that shows 15.1° [28]. The PI-LL mismatch from this study continued to slightly improve from six months to 12 months postop, and this may be due to the variability created by a single person taking the radiographic measurements. At 12 months postoperative, sagittal alignment parameters such as mean PI, PT, and SS from this study were shown to be within or very close to the typical ranges reported in the literature, 55.1° between 33° and 85°, 20.1° close to 7° to 19°, and 35.3° between 20° and 65°, respectively [29-31]. In short, radiographic results from this study show comparable radiographic corrections as compared to similar studies. This highlights the use of OLIF with PALLR and Ponte osteotomy for obtaining greater lordosis and coronal correction.

With respect to fusion rates, Zhu et al.’s meta-analysis showed a fusion rate of 94.1% [27]. This study’s result showed a 100% fusion rate (18/18) at 12 months postoperative. The high fusion rate from this study can be attributed to the use of titanium-based expandable spacers, which have shown higher fusion rates as compared to static spacers due to less impaction on vertebral endplates during insertion [19-21]. The mean neuroforaminal height increase of 1.8 mm was lower than other multi-level OLIF literature, reporting an average of approximately 5 mm [32]. The low foraminal height was most likely due to the use of Ponte osteotomy for correction of sagittal alignment, which requires the need to resect parts of the posterior spinal elements. This also explains the large statistically significant mean disc height increase of 4.1 mm in this study, which was on the upper end of the range (0.31-4.47 mm) reported in a meta-analysis by Zhang et al. on OLIF [33]. It is noteworthy to mention that the use of a spacer in the disc space allowed for the improved maintenance of the disc height up to 12 months postoperative.

PRO data showed statistically significant improvements in the average VAS back pain score of 21 mm and SF-12 of 5.2 points at the final follow-up. The average ODI also improved by 4.1% between the preoperative and final follow-up. Mean changes in VAS back pain and ODI at final follow-up were lower than those reported in a meta-analysis on ASD, with a mean change of 51 mm for VAS and 32.3% for ODI [34]. There are two main reasons for small changes in PRO values in the current study. First, the average preoperative VAS (61 mm) and ODI (27.1%) scores were relatively low compared to the meta-analysis’ preoperative VAS (73 mm) and ODI (52.7%) [35]. Second, this study involved a second-day posterior open surgery, where Ponte osteotomy was performed to provide additional sagittal correction. It is a more intensive overall surgery as compared to the meta-analysis, in which a majority of the studies did not include osteotomy. It is important to note that 50% (9/18) of the patients achieved MCID for VAS (i.e., ΔVAS ≥ 2) by the 12-month follow-up. A majority of patients in this cohort displayed clinically important changes in key a PRO parameter when performing multi-level PALLR ATP-LIF along with Ponte osteotomy in a two-stage surgery.

In summary, this study provides a complete clinical overview of a potential alternative technique for treating ASD patients. It also shows that the use of expandable spacers may provide an added benefit in terms of improving fusion rates, restoring disc heights, and positioning within the disc space (enabling coronal alignment).

Limitations and future directions

A limitation of this study was that there was a disparity in the response rate of patients to different PROMs. For example, out of 20 patients enrolled at the beginning of the study, 18 patients responded to VAS back pain, whereas only 10 patients responded for ODI and 9 patients for SF-12 at the 12-month follow-up visit. This study also had a minimum follow-up period of only 12 months, a longer follow-up period will provide better insights on long-term complications, which are expected in ASD cases. Another limitation was that it is a single surgeon experience, with a relatively small sample size. Future directions would be to replicate this study with a large patient group treated by different surgeons with a longer follow-up period. This may help formulate conclusions on the use of expandable interbody spacers for ATP-LIF for minimally invasive anterior column reconstruction to treat ASD.

## Conclusions

This study investigated the clinical use of expandable spacers in multi-level two-stage surgery for treating patients with ASD. ATP-LIF allows for less anatomical disruption, thereby providing a minimally invasive alternative for deformity surgery. It also permits PALLR, which complements the lordosis correction provided by the Ponte osteotomy. The surgical, radiographic, and PROs demonstrated that this type of surgery is a viable alternative to treat ASD. Overall, the patients in this study showed improvements in radiographic and PROs as compared to the preoperative baseline. Specifically, the high fusion rate, statistically significant changes seen in disc height increases, PI-LL mismatch correction, VAS back pain score reduction, and SF-12 improvement at final follow-up are supportive of these results. In addition, there were no intraoperative complications, and the postoperative complication rate was similar to that of other deformity studies and was lower than pedicle subtraction osteotomy. Despite the positive initial findings, more data are needed to evaluate this technique, including additional studies with larger sample sizes and longer follow-up periods.
